# Monitoring Community Pharmacist's Quality of Care: A feasibility study of using pharmacy claims data to assess performance

**DOI:** 10.1186/1472-6963-11-12

**Published:** 2011-01-18

**Authors:** Nancy Winslade, Laurel Taylor, Sherry Shi, Lambert Schuwirth, Cees Van der Vleuten, Robyn Tamblyn

**Affiliations:** 1 Department of Medicine, Faculty of Medicine, McGill University, 1140 Pine Avenue West, H3A 1A3, Montreal, Quebec, Canada; 2 Canadian Patient Safety Institute, 1150 Cyrville Road, Suite 410, Ottawa, K1J 7S9, Ontario, Canada; 3 Department of Educational Development and Research, Faculty of Health, Medicine and Life Sciences, University of Maastricht, PO Box 616, 6200 MD, Maastricht, The Netherlands

## Abstract

**Background:**

Public pressure has increasingly emphasized the need to ensure the continuing quality of care provided by health professionals over their careers. Health profession's regulatory authorities, mandated to be publicly accountable for safe and effective care, are revising their quality assurance programs to focus on regular evaluations of practitioner performance. New methods for routine screening of performance are required and the use of administrative data for measuring performance on quality of care indicators has been suggested as one attractive option. Preliminary studies have shown that community pharmacy claims databases contain the information required to operationalize quality of care indicators. The purpose of this project was to determine the feasibility of routine use of information from these databases by regulatory authorities to screen the quality of care provided at community pharmacies.

**Methods:**

Information from the Canadian province of Quebec's medication insurance program provided data on prescriptions dispensed in 2002 by more than 5000 pharmacists in 1799 community pharmacies. Pharmacy-specific performance rates were calculated on four quality of care indicators: two safety indicators (dispensing of contra-indicated benzodiazepines to seniors and dispensing of nonselective beta-blockers to patients with respiratory disease) and two effectiveness indicators (dispensing asthma or hypertension medications to non-compliant patients). Descriptive statistics were used to summarize performance.

**Results:**

Reliable estimates of performance could be obtained for more than 90% of pharmacies. The average rate of dispensing was 4.3% (range 0 - 42.5%) for contra-indicated benzodiazepines, 15.2% (range 0 - 100%) for nonselective beta-blockers to respiratory patients, 10.7% (range 0 - 70%) for hypertension medications to noncompliant patients, and 43.3% (0 - 91.6%) for short-acting beta-agonists in over-use situations. There were modest correlations in performance across the four indicators. Nine pharmacies (0.5%) performed in the lowest quartile in all four of the indicators, and 5.3% (n = 95) performed in the lowest quartile on three of four indicators.

**Conclusions:**

Routinely collected pharmacy claims data can be used to monitor indicators of the quality of care provided in community pharmacies, and may be useful in future to identify underperforming pharmacists, measure the impact of policy changes and determine predictors of best practices.

## Background

Although medications are a mainstay of modern medical treatment, their use is associated with both benefit and harm [1-3]. Preventable drug-related morbidity and mortality (DRM) now accounts for 8% to 23% of hospital admissions in seniors [4-6]. In North America in the early 1990's, professional pharmacy organizations recognized the extent of this problem and recommended that pharmacists expand their role to include a focus on minimizing preventable DRM [7-9]. This recommendation was based on the assertion that pharmacists are both educated to manage patients' drug-therapy problems and readily accessible within the majority of communities in North America. Indeed, the average Canadian adult visits a community pharmacy once or twice per month [10]. This situation provides an ideal opportunity for community pharmacists to minimize patient's preventable DRM.

As pharmacists have incorporated this role of minimizing DRM into their practice, university-based educational programs have been modified to emphasize these responsibilities and standards of practice have been revised [11,12]. Increasing public emphasis on the need for assurance that health practitioners remain competent to provide quality care through out their careers has lead pharmacy regulatory authorities to update their programs for monitoring pharmacist's performance to ensure compliance with these revised standards of practice [personal communications Quebec Order of Pharmacists and Nova Scotia College of Pharmacists]. These quality assurance programs are being updated based on modern frameworks for regulatory review that include proactive routine surveillance of practice performance, followed by more in-depth assessment of potentially under-performing practices [13-16]. Such frameworks acknowledge the collaborative nature of current health care provision and that the quality of care provided by the health care team is dependent on the quality of care provided by each team member [14,17]. These frameworks are appropriate for use in community pharmacies, where multiple pharmacists and pharmacy technicians contribute to the care and services provided to an individual patient [18,19]. Therefore, the proactive surveillance phase of regulatory programs recommended for community pharmacists measures performance on a pharmacy-specific basis rather than on a pharmacist-specific basis [14].

Indicators are being developed to conduct routine surveillance of the quality of care provided in community pharmacies [20]. To maximize efficiency, it has been recommended that performance on these indicators be assessed using pharmacy claims data that are routinely collected as part of daily provision of medications and services [14,20]. Preliminary studies have shown that the data required to measure performance on these indicators is available in pharmacy claims databases and that pharmacists find comparative performance information useful [21]. What remains to be evaluated is whether routine use of information from these databases to measure performance at community pharmacies is feasible. Feasibility can be determined by evaluating whether the database-derived performance measures meet the key requirements for use by regulatory authorities for quality assurance. Specifically, the target services need to be routinely provided at the majority of community pharmacies, care must be appropriately attributed, and there must be sufficient variability in performance across community pharmacies to warrant surveillance [22].

The purpose of this project was to determine the feasibility of routine use of information from these databases by regulatory authorities to screen the quality of care provided at community pharmacies.

## Methods

### Context

This study was conducted in the Canadian province of Quebec where the Order of Pharmacists, the governmental regulatory authority responsible for licensing pharmacists, monitors the performance of more than 5000 community pharmacists in 1800 community pharmacies. These pharmacists serve a population of 7.4 million patients, of whom approximately 50% receive government support for payment of their medications via the publicly-funded insurance program (Régie de l'assurance maladie du Québec - RAMQ). Similar to public and private insurance programs in the rest of the Canadian provinces and American states, medication-related data maintained by RAMQ for insured patients includes payments made for each dispensing of a reimbursable medication, the date, the name, strength, dosage form and quantity of the dispensed medication, and prescriber, pharmacist and pharmacy identification. Reimbursable medications include over 85% of medications available in Canada. In Quebec, medication supply policies support dispensing of chronic medications on a 30 day interval rather than encouraging a 90 day dispensing interval. Although this policy provides more opportunities for pharmacists to detect and intervene on medication use problems, dispensing on either a 30 day or 90 day interval provides regulatory authorities with the data required to measure performance on quality of care indicators.

### Study Population

To assess the quality of care provided at community pharmacies, we used a random sample of 1.4 million patients who received medications from community pharmacies between January and December 2002. In addition to the routine medication-related information, for each dispensing a unique anonymized identifier was provided by RAMQ for each patient, prescriber and dispensing pharmacy. These unique identifiers were used to develop a longitudinal prescription history for each patient and a practice population for each pharmacy that could be used to measure quality of performance on each indicator on a pharmacy-specific basis. Ethics approval was obtained from the McGill Faculty of Medicine Institutional Review Board.

### Assessing Feasibility

The feasibility of using pharmacy claims data to evaluate performance was assessed by calculating pharmacy-specific performance on four quality of care indicators and determining whether results met requirements for use for high-stakes decisions by regulatory authorities [22]. Specifically we evaluated the proportion of community pharmacies where provision of services was frequent enough to allow reliable assessment on the performance indicators, the variability of performance across pharmacies and the proportion of pharmacies where performance was systematically poor on multiple indicators, indicating a need for further evaluation.

### Selection and Measurement of Indicators

The Pharmacy Quality Alliance (PQA) in the United States has developed quality indicators for conditions where there are widely documented medication use problems, including noncompliance with anti-hypertensive medications, over-use of rescue inhalers in the treatment of asthma and use of contra-indicated/high risk medications [20]. We used similar indicators to assess performance at community pharmacies in Quebec. Two of the indicators measured safety (the use of high risk medications) and two represented effectiveness of chronic treatments (hypertension and asthma)(Table 1). To produce comparable measures of performance among pharmacies, we restricted the denominator for each indicator to the "at-risk" patient population. This at-risk population was defined as patients who were treated for the respective problem (e.g. hypertension) or who had been dispensed medications within a specific therapeutic class. For example, for the medication safety indicator evaluating the dispensing of flurazepam to seniors, the at-risk population was defined as 'all seniors who were dispensed a benzodiazepine from the pharmacy' and not as 'all seniors dispensed a medication from the pharmacy' as most would not be using a benzodiazepine and would, therefore, not be at-risk. Details of the at-risk population used for each indicator are found in Table 1.

**Table 1:** *Quality of Care *Indicators for Community Pharmacists

Indicator	Definition
**Medication Safety**	

Beta Blockers in Respiratory Patients	
*Recommended Care: *Patients receiving a SABA should **not **receive nonselective beta-blockers (NSBB)	*Measure: *proportion of dispensings of beta-blockers to patients dispensed SABA that are for NSBB*
	*Denominator: *# of dispensings of oral beta-blockers (selective or nonselective) to patients who received a SABA from any pharmacy within the previous 6 months (including dispensing on the same day)
	*Numerator: *# of dispensings of oral NSBB to patients who were dispensed a SABA from any pharmacy within the previous 6 months (including dispensing on the same day)
Benzodiazepines in the Elderly	
*Recommended Care: *seniors requiring a benzodiazepine should **not **receive long acting agents (flurazepam)	*Measure: *proportion of dispensings of benzodiazepines to patients >65 years old that are for flurazepam*
	*Denominator: *# of dispensings of benzodiazepines to patients >65 years old
	*Numerator: *# of dispensings of flurazepam to patients >65 years old
**Medication Effectiveness**	
Over-use of Asthma Medications	
*Recommended Care: *patients should **not **use more than 250 doses of SABA in a 90 day period	*Measure: *proportion of dispensings of SABA that are provided to patients who had used more than 250 doses of these rescue medications over the previous 90 days*
	*Denominator: *# of dispensings of inhaled SABA
	*Numerator: *# of dispensings of an inhaled SABA that were provided to a patient who had been dispensed more than 250 doses of this same SABA from any pharmacy within the previous 90 days
Under-use of Anti-hypertensives	
*Recommended Care: *patients should take at least 80% of their medication prescribed for hypertension	*Measure: *proportion of dispensings of anti-hypertensive medications that are provided to patients who had received <80% of their required supply of antihypertensive medications over the previous 90 days*
	*Denominator: *# of dispensings of any blood pressure medications
	*Numerator: *# of dispensings of any blood pressure medications that were to patients who had received <80% of the prescribed amount of any blood pressure medications over the previous 90 days from any pharmacy

* The lower the score on the *Quality of Care *indicators the better the performance.

Number of dispensings was counted in the numerator and denominator because the number of dispensings provided the best measure of the opportunity for community pharmacists to detect and intervene on medication-use problems. For example, for the indicator evaluating non-compliance with anti-hypertensive medications, pharmacists had the opportunity to detect non-compliance each time a patient returned to the pharmacy for a refill of his/her medication. Therefore, each dispensing to a non-compliant patient represented an opportunity for intervention by the pharmacist.

Pharmacy-specific performance on each quality indicator was calculated using dispensings of medications from all community pharmacies. All dispensings were included because pharmacists are expected to inquire about all prescription therapies including dispensings from other pharmacies. Operationally this meant that for the dispensing of concomitant non-selective beta-blockers and inhaled short acting beta agonists (SABA), the medications could be dispensed from two different pharmacies and the performance was attributed to the pharmacy dispensing the non-selective beta-blocker to the patient. For the over-use of SABA and non-compliance with anti-hypertensive medications, all SABA's and anti-hypertensives dispensed to the patient from all pharmacies were counted. As medication discontinuation and switching are not well documented in databases and are common in management of hypertension, the inclusion of all anti-hypertensives allowed us to eliminate the problems of over-estimation of non-compliance due to switching or changing dose of anti-hypertensive medications.

To ensure stable estimates of performance we required pharmacists at each pharmacy to dispense the relevant medication to the at-risk population (i.e. denominator group) five or more times over the one year period [23]. Such cut offs are recommended for use in measures of physician performance to improve the reliability of estimated performance while ensuring that potential poor-performers who provide a low volume of service are not excluded from evaluations [22,24].

### Data Analysis

To evaluate whether the performance on the quality indicator met the requirements established by regulatory authorities, we first determined the number of pharmacies where pharmacists provided the service frequently enough for reliable performance estimates to be calculated. We then used univariate statistics and the coefficient of variation to examine the distribution of the quality of performance across pharmacies. To determine if performance on one indicator influenced performance on another indicator, we estimated the Spearman Rank Order Correlation among indicators using the pharmacy as the unit of analysis. To estimate the proportion of pharmacies that would be in the worst-performing quartile in all indicators, and would likely be targeted for regulatory review, we cross tabulated performance by quartile on the four performance indicators.

## Results

Our sample included 1,427,325 patients with an average age of 50 and a predominance of women (Table 2). Patients were dispensed on average 6.2 different medications per year, receiving these medications via, on average, 37.9 dispensings per year. The mean number of pharmacies visited per patient per year was 1.6, with 61.4% of patients using one pharmacy exclusively over the study period. Although within our study population the greatest number of patients demonstrated under-use of anti-hypertensive medications (42.5%, 220,179 of 517,656 at-risk patients under-used anti-hypertensives), patients taking SABA were more likely to demonstrate inappropriate use of these medications (57.0%, 39,895 of 70,021 at-risk patients over-used SABA).

**Table 2:** Characteristics of Pharmacy Practice Population (N = 1,427,325 patients)

Patient Characteristics		
**Patient Demographics**	**N**	**%**
Female	849,989	59.6 ^†^
	**Mean**	**SD**
Age	50.0	24.2
**Medication use**	**Mean**	**SD**
Medications per year ^‡^	6.2	5.2
Dispensings per year ^§^	37.9	64.3
**Health Services Use**	**Mean**	**SD**
Prescribing physicians per year	2.7	2.0
Dispensing pharmacies per year	1.6	0.95
	**N**	**%**
Patients receiving all medications & services from a single pharmacy per year	877,038	61.4^†^
**Population Prevalence of Quality of Care Problems**	**N (N at-risk)**	% **
**Medication Safety Indicators**		
Seniors receiving flurazepam	11,453 (196,774)	5.8
Patients receiving non-selective beta blockers and SABA	5,200 (33,058)	15.7
**Medication Effectiveness Indicators**		
Patients over-using SABA	39,895 (70,021)	57.0
Patients under-using anti-hypertensives	220,179 (517,656)	42.5

† Percent of 1,427,325 patients

‡ Calculated by determining the average of the total number of medications per patient per year.

§ Total number of dispensings in one year period is 54,045,097.

** Percent of the population at-risk

### Breadth and Frequency of Service Provision

The majority of pharmacies provided the services for the four indicators frequently enough to be included in the performance calculation (Table 3). The indicator that excluded the greatest number of pharmacies was the indicator measuring the concomitant use of SABA and non-selective beta-blockers, reflecting the low prevalence of at-risk patients in the population (Table 2). For the indicator related to under-use of anti-hypertensive medications, virtually all pharmacies met the cut-off criteria of having dispensed anti-hypertensive medications five or more times over the study period. No pharmacy was excluded from the performance calculation on all four indicators.

**Table 3:** Pharmacy-Specific Performance on *Quality of Care *Indicators

	**Breadth/Frequency of Performance**				**Pharmacy-Specific Performance on *Quality of Care *Indicators**			
	
	**Pharmacies dispensing 5 or more times per year**		**Dispensings per year (N = 54,045,097)**		**Indicator**	**Mean Annual Pharmacy-specific Rate of Inappropriate Dispensings**		
	
	N	% ^††^	N	% ^‡‡^		Mean	SD	Range
**Medication Safety**								
Dispensings of benzodiazepines to seniors	1763	98.0	1,885,484	3.5	Seniors receiving flurazepam	4.3 ^§§^	3.1	0-42.5
Dispensings of beta-blockers to patients taking SABA	1730	96.2	398,177	0.7	Patients receiving non-selective beta blockers and SABA	15.2 ***	11.1	0-100
**Medication Effectiveness**								
Dispensings of SABA	1775	98.7	527,955	1.0	Patients over-using SABA	43.3 ^†††^	12.4	0-91.6
Dispensing of anti-hypertensive medications	1793	99.7	10,838,986	20.1	Patients under-using hypertension medications	10.7 ^‡‡‡^	4.0	0-70.0

^†† ^Of all pharmacies dispensing medications in our sample (1799)

^‡‡ ^Of all dispensings of medications to our population during 2002 (54,045,097)

^§§ ^Median annual dispensing rate for flurazepam is 4.0%, interquartile range 2.5%-5.7%

*** Median annual dispensing rate for SABA and non-selective beta-blockers is 14.2%, interquartile range 8.3%-20.4%

^††† ^Median annual dispensing rate for SABA over-use is 43.6%, interquartile range is 36.4%-50.7%

^‡‡‡ ^Median annual dispensing rate for HTN under-use is 10.2%, interquartile range is 8.4%-12.2%.

### Overall quality and variability in service provision

Overall the quality of performance was better for the medication-safety indicators than for the medication-effectiveness indicators (Table 3). The worst performance on the four quality of care indicators was exhibited for the SABA over-use indicator. On average, across all pharmacies, 43.3% dispensings of SABA were to patients who had over-used these medications within the previous 90 days. Performance on the medication-safety indicator measuring pharmacist dispensing of flurazepam to seniors was the best with, on average, 4.3 percent of benzodiazepine dispensings to seniors being for flurazepam.

**Figure 1:**
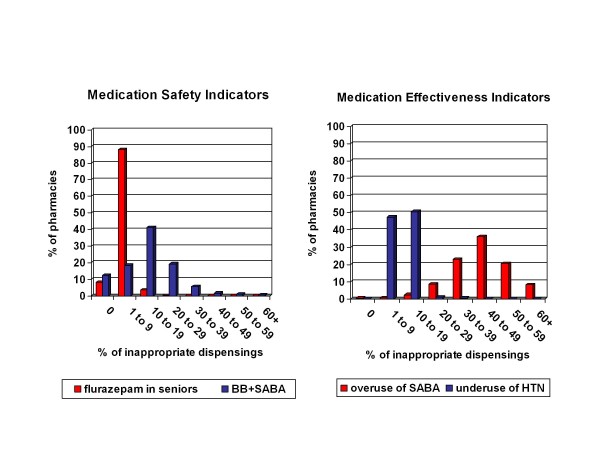
**Distribution of Pharmacies by Performance on Safety and Effectiveness Indicators as Measured by Pharmacy Specific Percent of Inappropriate Dispensings**.

Variability of performance across the community pharmacies on each of the indicators is also provided in Table 3. Based on the coefficient of variation (CV), performance on the medication-safety indicators demonstrated the greatest variability. Across pharmacies the rate of inappropriate dispensing ranged from 0% to 42.5% for flurazepam with a CV of 0.72, and 0% to 100% for nonselective beta-blockers and SABA (CV = 0.73)(Figure 1). In comparison, for medication-effectiveness indicators, the CVs of the two indicators were lower (0.29 for SABA over-use and 0.37 for anti-hypertensive under-use).

### Performance across indicators

Among the 1799 pharmacies, 86% provided a sufficient number of services to assess performance on all four indicators. The rank order correlation among indicators was modest, varying from -0.01 (nonselective beta-blockers concomitant with SABA and over-use of SABA) to a high of -0.13 (over-use of SABA and under-use of anti-hypertensive medications).

Nine pharmacies performed in the lowest quartile on all four indicators, with an additional 95 pharmacies performing in the lowest quartile on three of four indicators. These pharmacies represent those that, in all likelihood, would be targeted for further evaluation by regulatory authorities. By contrast, three pharmacies were in the top quartile for all four indicators with another 79 pharmacies in the top quartile for three of four indicators. These pharmacies would be useful to determine predictors of best practice.

## Discussion

This study evaluated all 1800 community pharmacists providing medications to 1.4 million people within the jurisdiction of one regulatory authority in Canada. We found that performance at community pharmacies could be measured on four quality of care indicators using pharmacy claims data. Over 85% of pharmacies provided services frequently enough to be reliably assessed on all four indicators. Proactive surveillance documented substantial variability in performance, with the best performance demonstrated on the indicator measuring rates of dispensing of contra-indicated benzodiazepines to the elderly, and the worst performance demonstrated on the dispensing of SABA to patients who were over-using this medication. Pharmacists at 5.3% (95/1799) of pharmacies underperformed on three of the four indicators.

This study is the first to be done in Canada that uses routinely available claims data to evaluate performance at community pharmacies. The PQA in the United States has completed a series of demonstration projects using administrative data from limited numbers of pharmacies (25 to 85 community pharmacies) to evaluate and report on pharmacy-based performance on quality indicators measuring similar medication use-problems [21]. Similar to our results, these projects indicate that pharmacy claims data contain the information necessary to measure a number of quality of care indicators and that performance varies across pharmacies. Direct comparison of our results with those from the demonstration projects is difficult, since limited performance results have been published. However, differences in results are anticipated as our project used different definitions for some indicators. For example, for SABA over-use we used the Canadian guidelines that define over-use as more than 250 doses in 90 days as prior research had shown that overuse of this magnitude is associated with an increased risk of asthma mortality [25-27]. In contrast, the PQA defined over-use as more than 600 doses in 90 days. As a result of these differences in quality definitions, performance results would be worse in our study as compared to in the demonstration projects. To assess differences in jurisdictions and pharmacy policies, it will be essential to use common metrics for quality measurement.

Comparison of our results with those from the demonstration projects is also difficult as the PQA indicators use patients as the unit of analysis where we elected to use dispensings as the unit of analysis. Although number of patients could have been used in our study, by doing so we would have been unable to detect differences in pharmacies where, for example, a patient was compliant for 11 of 12 dispensings as compared to a pharmacy where the patient was compliant for none of the 12 dispensings. If patients had been used as the unit of analysis, both patients would have been classified as non-compliant for the year and the performance of the two pharmacies considered to be equal. To create a more sensitive measure of performance we, therefore, elected to use the number of dispensings as the unit of analysis. Use of dispensings versus patients would also be anticipated to result in a greater variability in performance across pharmacies, whereas use of patients as the unit of analysis would provide an estimate of the impact of pharmacy services in the population. Both approaches will be useful to evaluate in the future.

The results of our work have significant implications for regulatory authorities as the approach of using claims data to measure performance on quality of care indicators appears to be feasible and reveals substantial variability in the quality of care provided at community pharmacies. With greater demand for public accountability for the quality of health professional practice, regulatory authorities are moving from a passive complaints-based process for monitoring to proactive quality assurance. Currently, pharmacy regulatory authorities have adopted very resource-intensive processes such as on-site inspections or competence testing for assessing the quality of practice [28-30]. Given limitations in availability of human resources, such processes are not able to ensure timely detection of potential performance problems that require further investigation. The ubiquitous availability of pharmacy claims data, and ultimately electronic health records, and the reliability of this data provides a new avenue for relatively inexpensive, proactive performance assessment by regulatory authorities [31]. The methodologies for performance monitoring using billing and electronic health record data are being developed in a number of sectors such as pay-for-performance [32]. Pharmacy regulatory authorities will be able to capitalize upon such advances while developing their performance assessment programs. These approaches may also be more acceptable to practitioners since they are based on actual performance data rather than artificial testing or inspection processes [16].

The main strengths of this study are the large and representative population of pharmacies studied and the measurement of quality indicators in comparable populations. Limitations that should be considered in interpreting the results are that only four indicators were evaluated that may not be representative of a pharmacy's quality of care, and the relationship between performance on the indicator and patient outcome was not ascertained. We were also missing data such as treatment indication, and patient age, sex and socioeconomic status, all of which could influence the types of drugs prescribed and their utilization. In future it would be preferable to retrieve information on these patient-related characteristics to statistically adjust for differences in clientele served by different pharmacies. In addition to these patient characteristics that may affect pharmacy-specific performance, pharmacy-level characteristics have also been shown to relate to the quality of care provided in community pharmacies [33]. Future work should evaluate the influence of these pharmacy-level characteristics, such as pharmacy location, dispensing volume and staffing, on the quality of care provided at community pharmacies.

Our results confirm that patients present to community pharmacies with evidence of well known medication use problems that are related to safe and effective use of medications. Patients at some pharmacies receive superior care and our methodologies could be used to evaluate the patient, pharmacy and prescriber-based characteristics that are associated with better care. There are many changes being made to the way services are being provided in community pharmacies such as regulation of pharmacy technicians, remote dispensing and deregulation of prescription medications. The utilization of pharmacy claims data provides unique opportunities to evaluate the influences of such policy changes.

## Conclusion

Routinely collected pharmacy claims data can be used to monitor indicators of quality of pharmacy services, and may be useful in future to identify potentially underperforming pharmacists, measure the impact of policy changes and to determine predictors of best practices.

## Competing interests

The authors declare that they have no competing interests.

## Authors' contributions

NW led the conception and design of the study, operationalized the indicators, led the analysis and interpretation of the data, prepared the manuscript and gave final approval of the version of the paper to be published. LT contributed to the conception of the indicators and their operationalization and revised the manuscript. SS completed the data analysis. LS and CVV contributed to the conception of the study and analysis of results and reviewed the manuscript for psychometric accuracy and applicability to assessment of practicing health care professionals. RT provided expertise in study design and operationalization of indicators, analysis and interpretation of the data and preparation of the manuscript. All authors read and approved the final manuscript.

## Pre-publication history

The pre-publication history for this paper can be accessed here:

http://www.biomedcentral.com/1472-6963/11/12/prepub
